# Surface topologies and self interactions in reactive and nonreactive Richtmyer–Meshkov instability

**DOI:** 10.1038/s41598-023-27904-w

**Published:** 2023-01-16

**Authors:** Maximilian Bambauer, Josef Hasslberger, Gulcan Ozel-Erol, Nilanjan Chakraborty, Markus Klein

**Affiliations:** 1 Department of Aerospace Engineering, University of the Bundeswehr Munich, 85577 Neubiberg, Germany; 2 School of Engineering, Newcastle University, Newcastle upon Tyne, NE1 7RU UK

**Keywords:** Fluid dynamics, Mechanical engineering

## Abstract

The reactive Richtmyer–Meshkov instability (RMI) exhibits strong wrinkling of a reactive flame front after an interaction with a shock wave. High levels of deformation and wrinkling can cause the flame surface to intersect with itself, leading to the events of flame self interactions (FSI). As FSI can have a significant influence on the development and topology of the flame surface, it should be considered an important factor affecting the burning characteristics of the flame. The topological structure and statistics of FSI are analyzed using data from high-fidelity simulations of a planar shock wave interacting with a statistically planar hydrogen/air flame for stoichiometric, lean and nonreactive gas mixtures. FSI events are detected by searching for critical points in the field of the reaction progress variable *c* and divided into the following topological categories: burned gas mixture pocket (BP), unburned gas mixture pocket (UP), tunnel formation (TF) and tunnel closure (TC). It is found that reactivity and flame thickness are decisive factors, influencing the frequency and topological distribution of the detected FSI events. While in early RMI-stages the FSI is found to be mainly dependent on the flame thickness, later stages are heavily influenced by the reactivity, as high reactivity quickly burns out emerging wrinkled structures (in the stoichiometric case) leading to massively reduced levels of FSI. The findings are further supported by the results from the nonreactive case, which at later stages of the RMI closely resembles the less reactive lean case. Analysis of the topology distribution over time and conditioned over *c*, reveals further differences between the lean and stoichiometric case, as the strong wrinkling and mixing encountered with the lean case facilitates the build up of many pocket-type and tunnel-type interactions throughout the wrinkled flame front. For the stoichiometric case, mainly tunnel-type and unburned pocket topologies are found in the narrow flame funnels extending into the burned gas.

## Introduction

**Figure 1:**
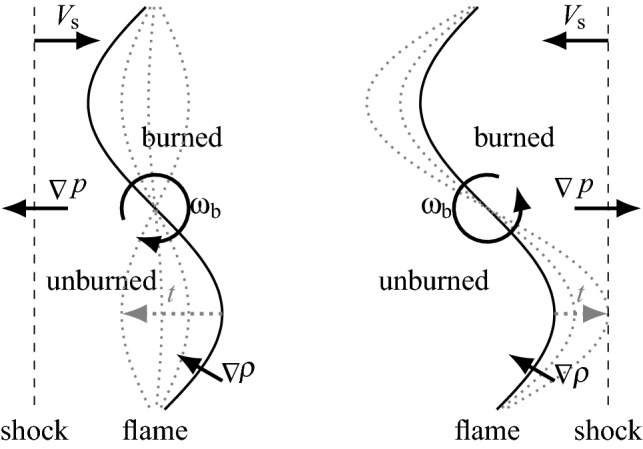
Schematic of the Richtmyer–Meshkov mechanism. Left: Shock wave propagating at speed $V_{\text {s}}$ from unburned to burned gas, causing the phase reversal effect. Right: Shock wave propagating from burned to unburned gas, amplifying the initial disturbance.

The production of baroclinic torque and subsequent deformation of the flame surface, caused by the interaction of a shock wave and a flame is referred to as the reactive Richtmyer–Meshkov instability (RMI)^1,2^. Figure 1 depicts the principal mechanism of the RMI,which is caused by the misalignment of the density gradient $\nabla \rho$ across the flame (or general interface between two fluids of different density) and the pressure gradient $\nabla p$ across a shock wave. The misalignment results in the production of baroclinic torque $\dot{\mathbf {\omega }}_{\text {b}}$, which is given by the following equation

1 $$\begin{aligned} \dot{\mathbf {\omega }}_{\text {b}} = \frac{\nabla \rho \times \nabla p}{\rho ^2} ~. \end{aligned}$$

As shown in Fig. 1 the baroclinic torque causes the growth of surface disturbances, with the special case of a phase reversal (Fig. 1 left) when the shock travels from the heavy (unburned) into the light (burned) gas. The highly non-linear nature of the RMI (especially in later stages) poses a major challenge towards its modelling^3^. An extensive survey of experimental observations, numerical simulations, modelling efforts and technical applications has been collected in a variety of in depth review literature^4–7^. As implied by Eq. (1), the equivalence ratio (affecting $\nabla \rho$), the shock Mach number (affecting $\nabla p$) and the initial flame perturbation (affecting the misalignment of $\nabla \rho$ and $\nabla p$) are key influencing factors in the development of the RMI.

The reactive RMI is a special case, where the heavy and light gas interface is represented by unburned (heavy) and burned (light) gas, which is separated by a flame. In essence, the reactive RMI is characterized by two competing effects. On the one hand, there is a significant increase in the integral reaction rate and turbulent flame speed^8,9^, due to the heavy wrinkling of the flame caused by the baroclinic torque. On the other hand, it has been shown in experiments^10^ and in numerical simulations^11^, that the reactivity itself can cause a decrease in flame wrinkling, due to reactive burnout of the developing wrinkled structures. In geometrically confined explosions, the RMI can be an important contributor to flame acceleration (FA) and deflagration to detonation transition (DDT), as the increased wrinkling and folding of the reactive flame front causes a rise in flame surface area and volume-integrated reaction rate^10^. For the closely related phenomenon of reactive shock-bubble interactions, experiments^12^ and numerical simulations^13–15^ have proven the RMI to be a major influence on self ignition, flame acceleration and transition to detonation. In industrial scale accident scenarios, the large spectrum of time and length scales required to accurately resolve the RMI can make the prediction of FA, DDT and overall explosion loads difficult^16^. Therefore, for large geometries, the usage of unsteady Reynolds averaged Navier Stokes (URANS) or Large Eddy Simulations (LES) is inevitable and the computationally expensive small scale RMI effects should be accounted for by suitable closures. In this context, well resolved high-fidelity simulations of the RMI in academic configurations can be of great use, as they contribute to the understanding of small scale effects. These insights can be utilized to create and improve modeling approaches, which are better suited to handle RMI specific effects in large scale (i.e. lower resolved) simulations.

In addition to the influence of flame wrinkling on surface generation, wrinkled flame surfaces can intersect and interact with each other, which potentially decreases the flame area. These flame self interaction (FSI) events can be detected during all stages of the RMI and can have a strong non-linear influence on the topology of the flame surface and therefore its burning characteristics. Previous studies^17,18^ have already shown that the equivalence ratio and reaction rate can have a significant effect on the development of the flame surface area during the RMI. The flame surface area was found to undergo several stages of growth, first linear and later nonlinear, where flame self interactions are suspected to be a primary influencing factor during the nonlinear stages of surface area growth and subsequent decay. The study of isosurface self interactions and topological structures via the analysis of critical points in scalar gradient fields was first introduced by Gibson^19^ in the context of turbulent scalar mixing. Two main methods of topology classification for the FSI events have been established. Dopazo et al.^20^ describes a method which utilizes local curvature to classify small-scale structures in terms of their mean and Gauss curvatures. An alternative characterization approach utilizes shape factors derived from the eigenvalues of the Hessian tensor^21,22^ after expansion of the gradient field around critical points (see Eq. 3). Both approaches are utilized in this work, with the former better describing the local (flame) geometry, whereas the latter describes the different types of self interaction phenomena. For turbulent premixed flames self interactions have been found to be an important factor, especially at high turbulence intensities^23^, locally altering the flame surface and affecting the overall burning rate^24–26^. This work aims to quantify and characterize the role of FSI during all stages of the RMI, using simulation data of shock-flame interactions in a homogeneous ${\text {H}}_2$/air mixture, including stoichiometric, lean and nonreactive cases. The FSI events are tracked and characterized by their topological structure using critical point theory^22^ and curvature statistics. The results include analysis of the temporal development of the FSI events, which are further characterized by their local topology and flame curvature.

## Results

**Figure 2:**
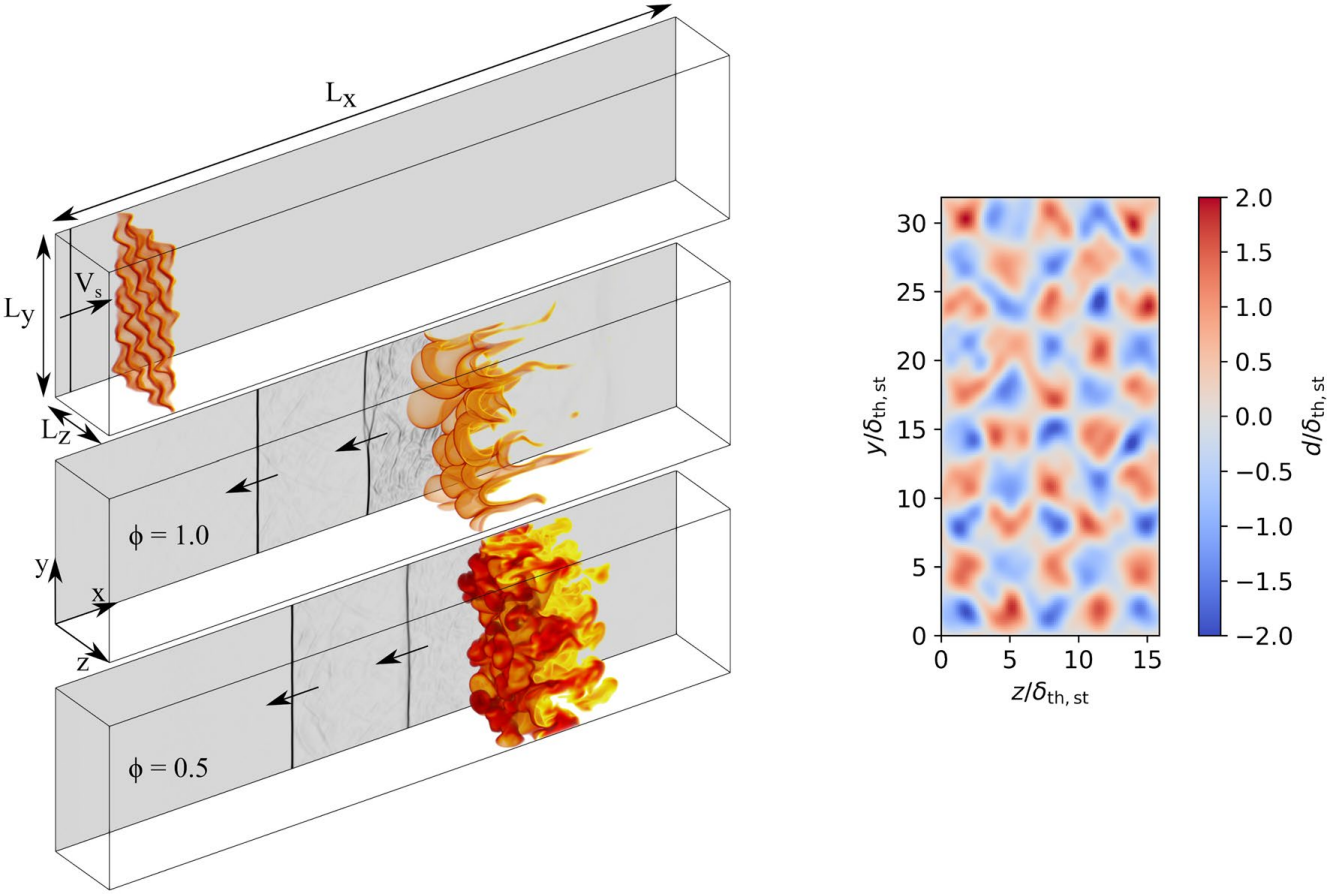
Left: Simulation setup (exemplary for $\phi =1.0$). At $t=0$ a planar shock wave is initialized with the velocity $V_{\text {s}}$ and propagates in positive x-direction towards the perturbed flame (top). Flame wrinkling after reshock interactions for $\phi =1.0$ (middle) and $\phi =0.5$ (bottom) at $t\times S_{\text {L,st}}/\delta _{\text {th,st}}=0.6$. Flame is shown as semitransparent iso-volume of varying opacity ($c\ge 0.15$/dark red and $c\le 0.85$/bright yellow). Grayscale slice of the pressure gradient magnitude (indicating the shock position) shown in background. Right: Normalized quasi-stochastic initial flame distortion in x-direction.

Figure 2 shows the simulation setup consisting of a planar shock wave at shock Mach number ${\text {Ma}}_{\text {s}}=V_{\text {s}}/a_0=1.5$ initially propagating in positive x-direction and a statistically planar ${\text {H}}_2$/air flame. With the heat capacity ratio $\gamma$, the specific gas constant $R_{\text {s}}$ and the initial temperature of the unburned gas mixture $T_0$, the reference speed of sound is defined as $a_0=\sqrt{\gamma R_{\text {s}} T_0}$. At $x=0$ a modified Navier–Stokes characteristics boundary condition (NSCBC) allows for local inflow and outflow of gases, while an adiabatic wall boundary condition is applied at $x=L_{\text {x}}$, resulting in shock reflections. Periodic boundary conditions are implemented in the *y* and *z* directions. The initial flame distortion is achieved by superimposing a single mode base oscillation with a quasi-stochastic multimode oscillation of smaller amplitudes^27^. The initial distortion in x-direction *d*(*y*, *z*) is calculated using the following function

2 $$\begin{aligned} d(y,z) = a_1 \sin (k_0 y) \sin (k_0 z) + a_2 \sum _{n=1}^{13} \sum _{m=3}^{15} a_{n,m} \sin (k_n y + \Phi _n) \sin (k_m z + \chi _m) ~, \end{aligned}$$

where $k_0=10\pi /L_{\text {y}}$ describes the base wavenumber and $k_n=2n\pi /L_{\text {y}}$ and $k_m=2m\pi /L_{\text {y}}$ are the distortion wavenumbers. The phases are given by $\Phi _n=\tan (n)$ and $\chi _{\text {m}}=\tan (m)$. To ensure sufficient resolution of the base and distortion perturbation, the base amplitude is set to $a_1=-1.25\delta _{\text {th,st}}$ and the distortion amplitudes are set to $a_2=-0.1a_1$ and $a_{n,m}=\sin (nm)/2$. The generic perturbation field generated from Eq. (2) is shown in Fig. 2 (right).

**Figure 3:**
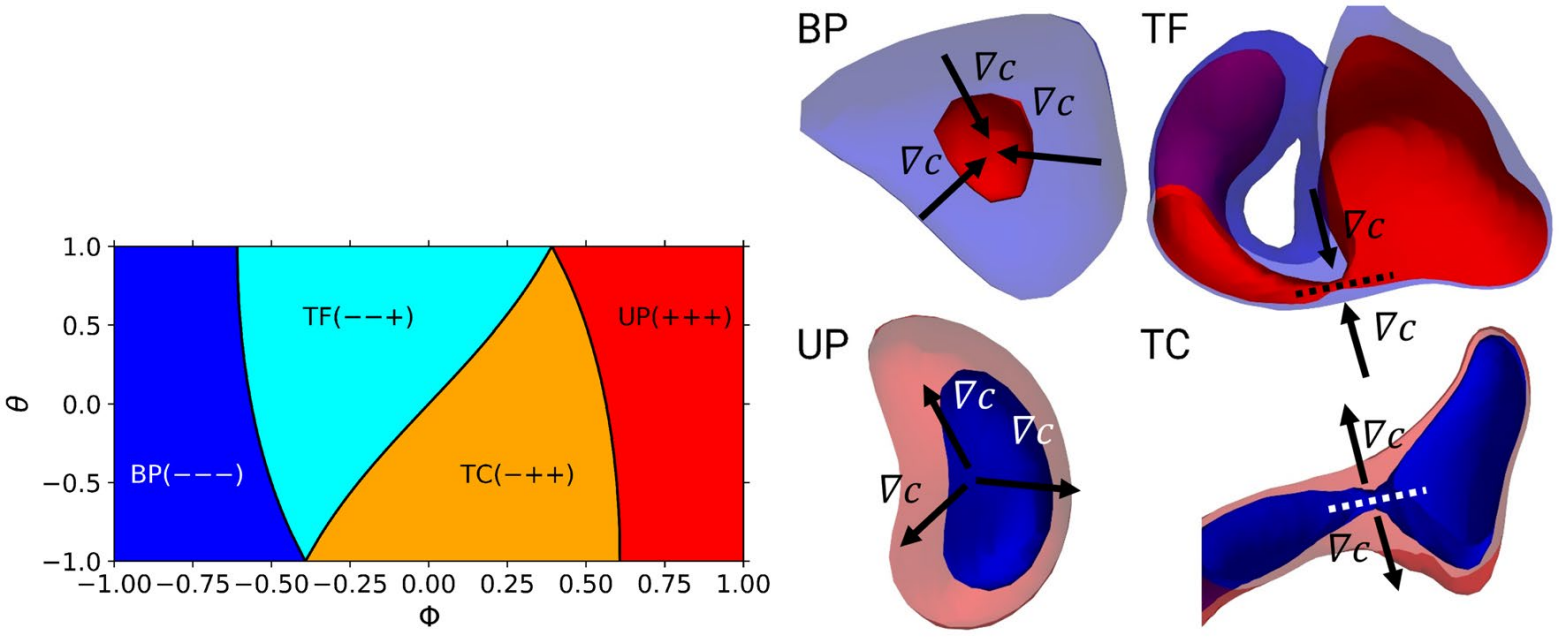
Topology types of the FSI (left) as characterized by the shape factors (burned pocket: BP, unburned pocket: UP, tunnel formation: TF, tunnel closure: TC). The black boundary lines between the colored areas denote sign changes in an eigenvalue of $\underline{{\underline{H}}}(c)$. Examples of FSI topologies (right), with blue isosurfaces denoting low values of *c* and red isosurfaces denoting high values of *c* (the enveloping isosurfaces are semitransparent). In addition, the gradient direction of *c* is shown schematically. The examples are taken from the lean case after the reshock.

Generally a FSI event can be defined as an isosurface of the reaction progress variable colliding with itself. The collision points can be detected by searching for critical points within the flame (see Griffiths et al.^22^ for the tracking methodology), where the gradient of the reaction progress variable vanishes. With $\nabla c = 0$, a Taylor series expansion around a critical point ${\textbf{a}}$ reduces to

3 $$\begin{aligned} c({\textbf{a}}+{\textbf{x}}) = c({\textbf{a}}) + \frac{{\textbf{x}}^T}{2}\underline{{\underline{H}}}(c({\textbf{a}})) {\textbf{x}} +\cdots ~, \end{aligned}$$

where the eigenvalues ($\lambda _1>\lambda _2>\lambda _3$) of the Hessian $\underline{{\underline{H}}}(c)$ describe the local topology of the self interactions^22^. By converting the eigenvalues $\lambda _i$ to spherical coordinates with the shape factors $\theta$ and $\varPhi$, the range of local FSI topologies can be described in a continuous two-dimensional domain.

4 $$\begin{aligned} \theta&= \frac{6}{\pi } \arctan \left( \frac{(\lambda _1-2\lambda _2+\lambda _3)}{\sqrt{6}(\lambda _1 -\lambda _3)/\sqrt{2}} \right) \end{aligned}$$

5 $$\begin{aligned} \varPhi&= \frac{2}{\pi } \arctan \left( \frac{(\lambda _1+\lambda _2+\lambda _3)\cos \left( \frac{\theta \pi }{6}\right) }{\sqrt{3}(\lambda _1-\lambda _3)/\sqrt{2}}\right) \end{aligned}$$

Figure 3 (left) shows the distribution of FSI topologies as defined by the normalized range of shape factors $\theta$ and $\varPhi$. The background colors denote different topologies, which are defined by the sign of the eigenvalues ($+$ or −) of $\underline{{\underline{H}}}(c)$, while the black boundary lines denote a sign change in these eigenvalues. From left to right the topologies can be categorized into burned mixture pocket (BP; $---$), tunnel formation (TF; $--+$), tunnel closure (TC; $-++$) and unburned mixture pocket (UP; $+++$). As shown by the examples in Fig. 3 (right) the BP topologies correspond to outward-propagating spherical regions of burned gas, likewise the UP topologies represent inward-propagating spherical regions of unburned gas. The TF (TC) topologies are slightly more complex, as they represent burned (unburned) cylindrical regions propagating away from (towards) a common axis. As the identification of FSI topologies is restricted to the flame, only critical points within the region $0.01\le c \le 0.99$ are taken into account.

**Figure 4:**
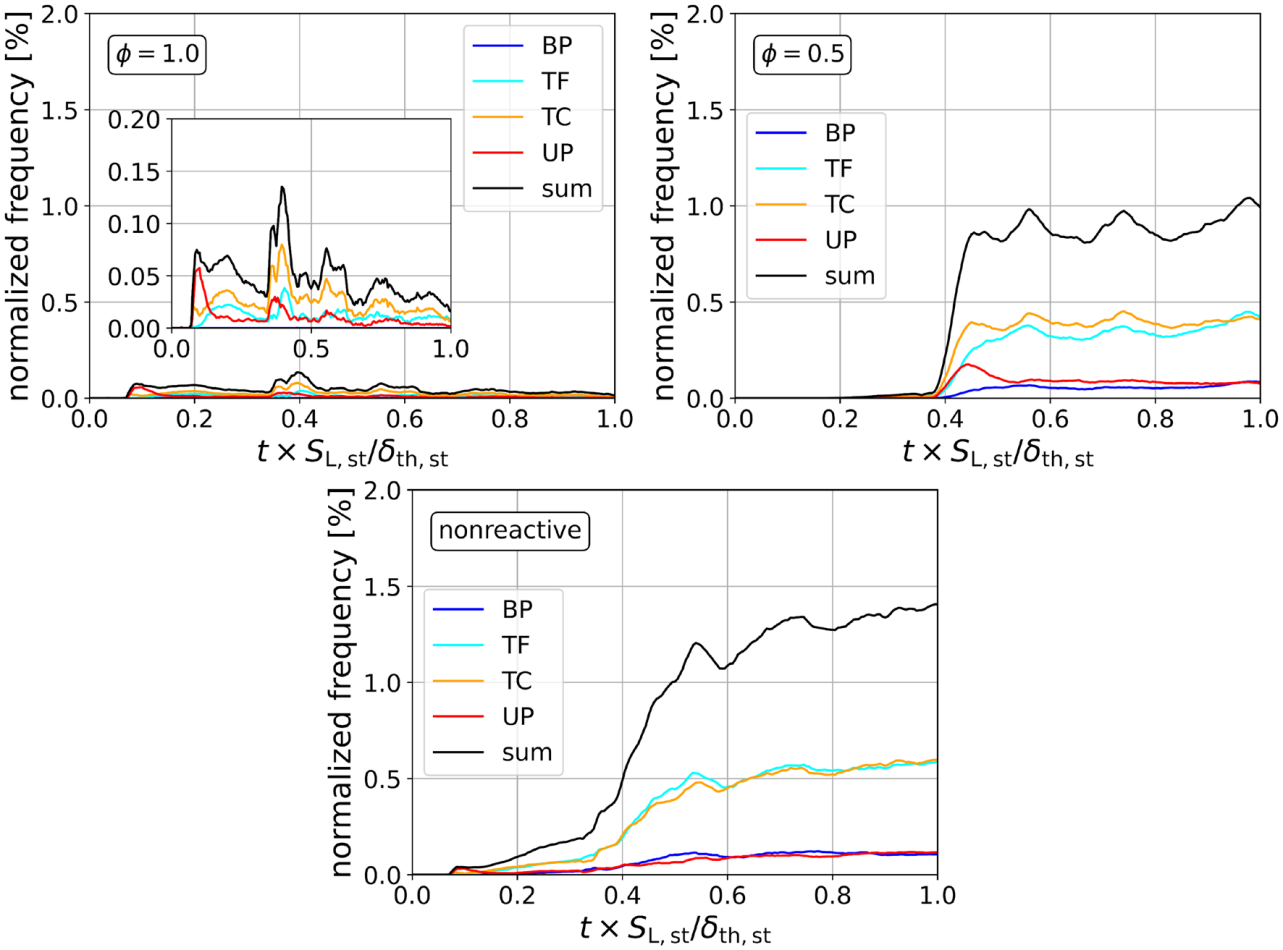
Normalized frequency of FSI topologies over normalized time for $\phi =1.0$, $\phi =0.5$ and nonreactive cases.

**Figure 5:**
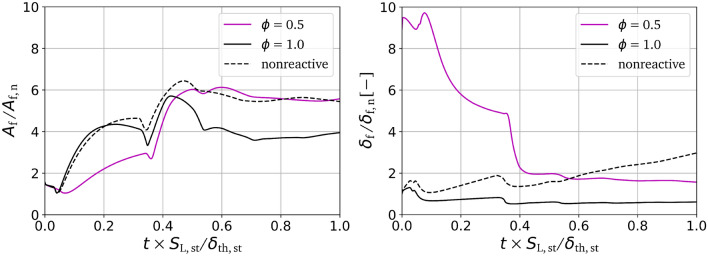
Normalized flame surface area (left) and normalized flame thickness (right) over normalized time for $\phi =1.0$, $\phi =0.5$ and nonreactive cases.

By extracting the critical points from the available simulation data for the $\phi =0.5,1.0$ and nonreactive cases following Griffiths et al.^22^, FSI events can be identified and the topology can be determined using the previously mentioned methods. Figure 4 shows the normalized frequency of detected FSI events over time. For normalization, the total frequency count is divided by the total flame volume $V_{\text {f}}$ at each given time step. As every detected FSI event corresponds to a cell within the flame, the normalized frequency is bounded between 0 and 1 and can be interpreted as the percentage of flame volume involved with FSI. In addition, Fig. 5 (right) shows the development of the normalized flame thickness over time for each case, as this is an important element in interpreting the behavior of the FSI. The flame thickness $\delta _{\text {f}} = V_{\text {f}}/A_{\text {f}}$ is defined as the ratio of flame volume $V_{\text {f}}$ to flame surface area $A_{\text {f}}$, where $A_{\text {f}}=\iiint _V |\nabla c|dV$ is based on the volume integral of the surface density function. The flame volume $V_{\text {f}}$ is calculated by summation of all grid points with $0.01 \le c \le 0.99$. The temporal evolution of the normalized flame surface area $A_{\text {f}}/A_{\text {f,n}}$ is shown in Fig. 5 (left). Choosing the channel cross-section for normalization $A_{\text {f,n}}=L_{\text {y}}L_{\text {z}}$, the normalized flame surface area can be interpreted as a wrinkling factor, which is of a pivotal importance for the closure of the reactive source term in under-resolved simulation approaches. The development of the normalized flame surface area can be described in two phases^18^. The first phase, following the initial shock flame interaction, is dominated by the effects of flame thickness, where the thinner stoichiometric flame is more susceptible to flame wrinkling than the thicker lean flame. In the second stage, the effects of reactivity become more important as the normalized flame surface area is reduced due to burnout of fresh-gas cusps in the stoichiometric case. In the lean case, the burnout effect is much less pronounced. However, there is still a slight reduction of flame surface area, possibly due to the influence of FSI.

Similar to the normalized flame surface area, the overall FSI development and topology distribution are found to be influenced by the flame thickness $\delta _{\text {f}}$ and the reactivity (as measured by $S_{\text {L}}$), which are both dependent on the equivalence ratio $\phi$. These factors cannot be seen as independent of each other as, for example, each shock-flame interaction strongly reduces the flame thickness over the course of the simulation. In general, shock-flame interactions will cause wrinkling of the flame, leading to increased amounts of self interaction. As a heavily wrinkled flame is more likely to interact with itself, an increased frequency of FSI detections is expected after shock interactions. This behavior can be qualitatively seen for both $\phi =1.0$ and $\phi =0.5$ (and nonreactive) cases. However, these cases are quantitatively different as the total normalized frequency count differs by a factor of 10–20, with $\phi =1.0$ at around $0.05\%$ and $\phi =0.5$ between 0.75 and $1\%$. This means that a significantly higher percentage of flame volume is associated with FSI in the lean case. For $\phi =1.0$, a distinct frequency peak can be seen at each moment of shock-flame interaction, which quickly diminishes after a short time frame. The overall low levels of FSI and the quick reduction of peaks seen in the stoichiometric case (Fig. 4), can be explained by its high reactivity, which causes a burnout of any emerging small wrinkled structures and prohibits further distortion of the wrinkled flame front. For $\phi =0.5$, the first shock interaction has a negligible effect on the build up of FSI, reaching even lower levels than the stoichiometric case, due to the initial high flame thickness making the flame more resistant to flame wrinkling. The following reshock causes a steep increase towards a maximum normalized frequency of about $1\%$. These high levels can be reached due to the increased amounts of wrinkling and mixing enabled by the low reactivity (see Fig. 2). The role of the reactivity is further emphasized when analyzing the nonreactive case. Here, the initial setup parameters resemble the $\phi =1.0$ case, but with the reaction rate of the progress variable ${\dot{\omega }}=0$. Initially, after interacting with the shock the first time, the behavior of the nonreactive case shows qualitative similarities with $\phi =1.0$. At later times, after the reshock, the behavior closely resembles to the $\phi =0.5$ case. This is due to the fact that the nonreactive case initially resembles the $\phi =1.0$ case both in flame thickness and thermodynamic properties, which in early stages dominates the FSI development. At later stages, the effect of the deactivated reactivity becomes more pronounced, as the increased levels of small scale wrinkling allow for more self interaction events to occur, similar to the comparatively less reactive fuel-lean case. While reactivity becomes important at later stages, the flame thickness $\delta _{\text {f}}$ strongly influences the self interaction behavior at early times, with $\delta _{\text {f}}$ being almost 10 times higher for $\phi =0.5$ than for $\phi =1.0$. Since the density gradient in a thick flame will generally be lower than for a thin flame, the wrinkling induced by the baroclinic torque will be less severe and therefore less self interactions will occur. At later times, the flame thickness is reduced by the shock-interactions (pressure increase and hence temperature increase), making the reactivity the dominant influencing factor.

In general, tunnel-type topologies dominate, although short peaks of UPs can be seen during shock-flame interactions in both cases. Similar observations have been made in the analysis of an open turbulent jet spray flame, where TCs and TFs are found to be the predominant topologies^28^. For $\phi =0.5$ and also in the nonreactive cases, an equilibrium state is reached, where the tunnel-type (pocket-type) topologies each make up $\approx 40\%$ ($\approx 10\%$) of total FSI topologies, which can be explained by the intermixing of small pockets of burned and unburned gas throughout the lean flame. For $\phi =1.0$, no BPs are detected with short peaks of UPs, TCs and TFs after each shock interaction.

**Figure 6:**
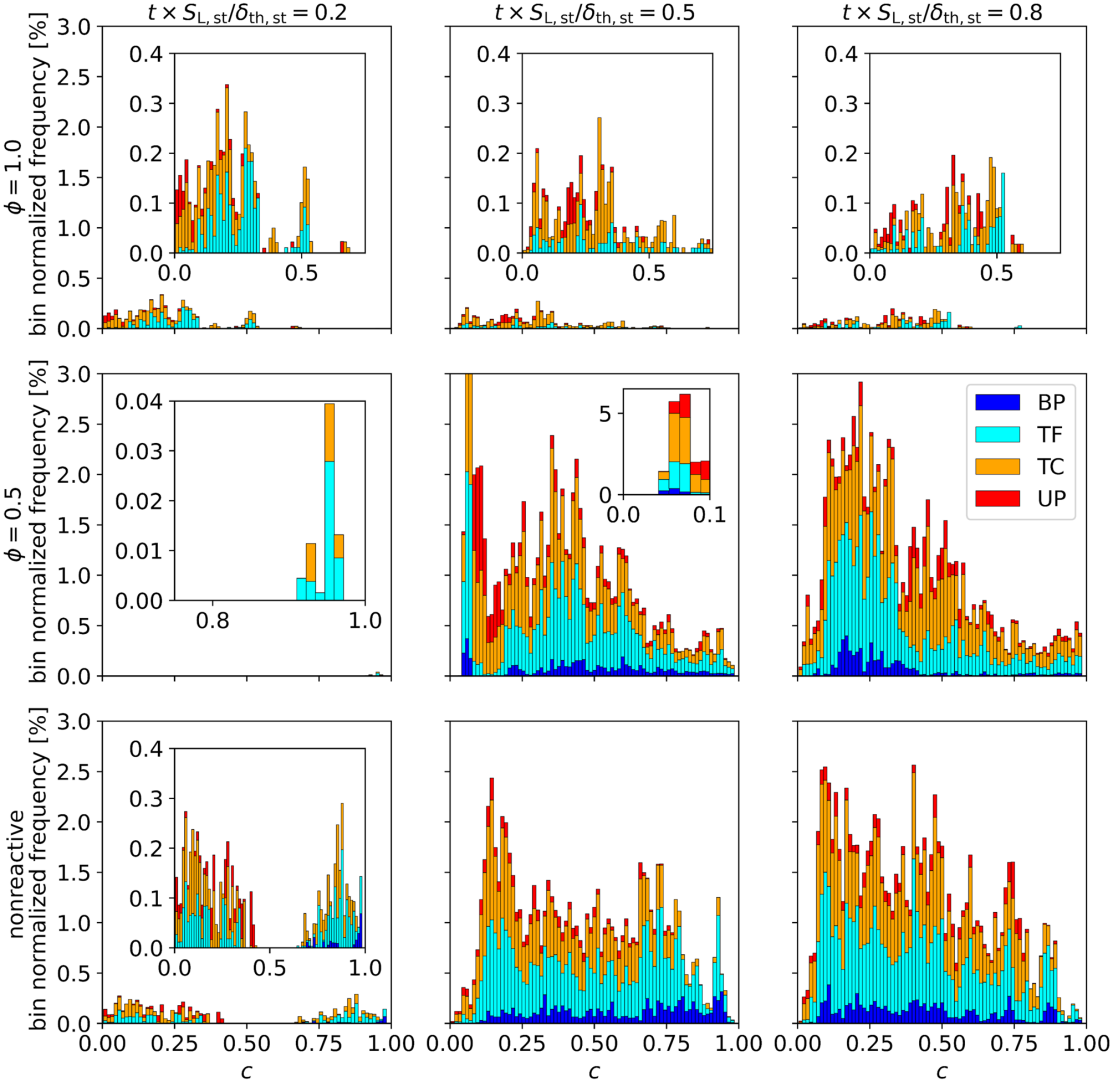
Histograms of the FSI topology counts (normalized with the respective bin volume) over *c* for the normalized times $t\times S_{\text {L,st}}/\delta _{\text {th,st}}=0.2,0.5,0.8$ (after first shock, directly and later after reshock) and the three cases $\phi =1.0$, $\phi =0.5$ and nonreactive.

An insight into the distribution of FSI topologies throughout the flame is presented in Fig. 6, showing histograms of the bin normalized frequency (normalized by the flame volume in each *c*-bin) conditioned over the reaction progress variable *c*. The times considered represent the distribution after the first shock interaction, directly after the reshock and the late-time behavior after the reshock. For $\phi =1.0$, the FSI events primarily take place in the region of $c<0.5$, which leads to mostly UP and TC type topologies. The overall low frequency of FSI events at $\phi =1.0$ makes an exact analysis difficult, as topology counts can show high fluctuations. Nonetheless, there is a slight tendency of the topology distribution to shift towards $c=0.5$, as time progresses. Due to the large flame thickness at $\phi =0.5$ (Fig. 5), only an insignificant amount of FSI are detected shortly after the first shock interaction. After the reshock, the interaction count increases rapidly and the topologies are well distributed over the whole *c*-range. The distribution of topologies across the entire flame front is again a sign of the strong mixing of burned and unburned gas, causing self interactions throughout all regions of the lean flame. Although less pronounced than for the $\phi =1.0$ case, there is a slight accumulation of FSI events in the region of $c<0.5$, which becomes more pronounced at later times. Similar to Fig. 4, the nonreactive case shows similarities to the stoichiometric case at early times, transitioning to a behavior resembling the lean case after the reshock. Interestingly, the nonreactive case shows a bimodal distribution after the initial shock-flame interaction with an additional accumulation (compared with $\phi =1.0$) of BPs and TFs around $c=0.9$. After the reshock, the topologies are distributed over *c* more uniformly than in the lean case, which is an indication that the deactivation of the reactive source term facilitates slightly better mixing of *c*.

**Figure 7:**
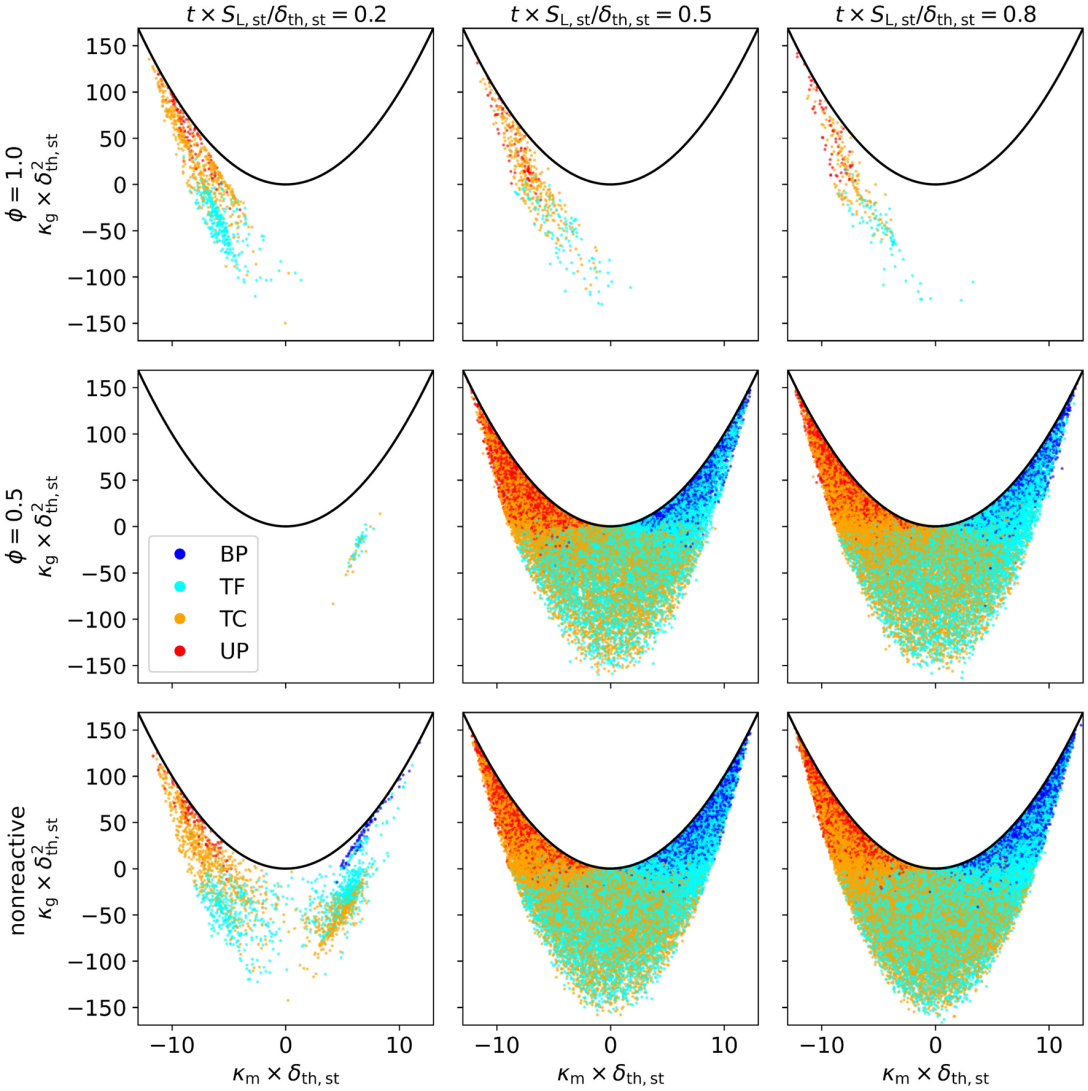
Scatter plot of FSI events on the normalized $\kappa _{\text {m}}-\kappa _{\text {g}}$ plane for the normalized times $t\times S_{\text {L,st}}/\delta _{\text {th,st}}=0.2,0.5,0.8$ (after first shock, directly and later after reshock) and the three cases $\phi =1.0$, $\phi =0.5$ and nonreactive. The FSI events are coloured by their respective topologies.

**Figure 8:**
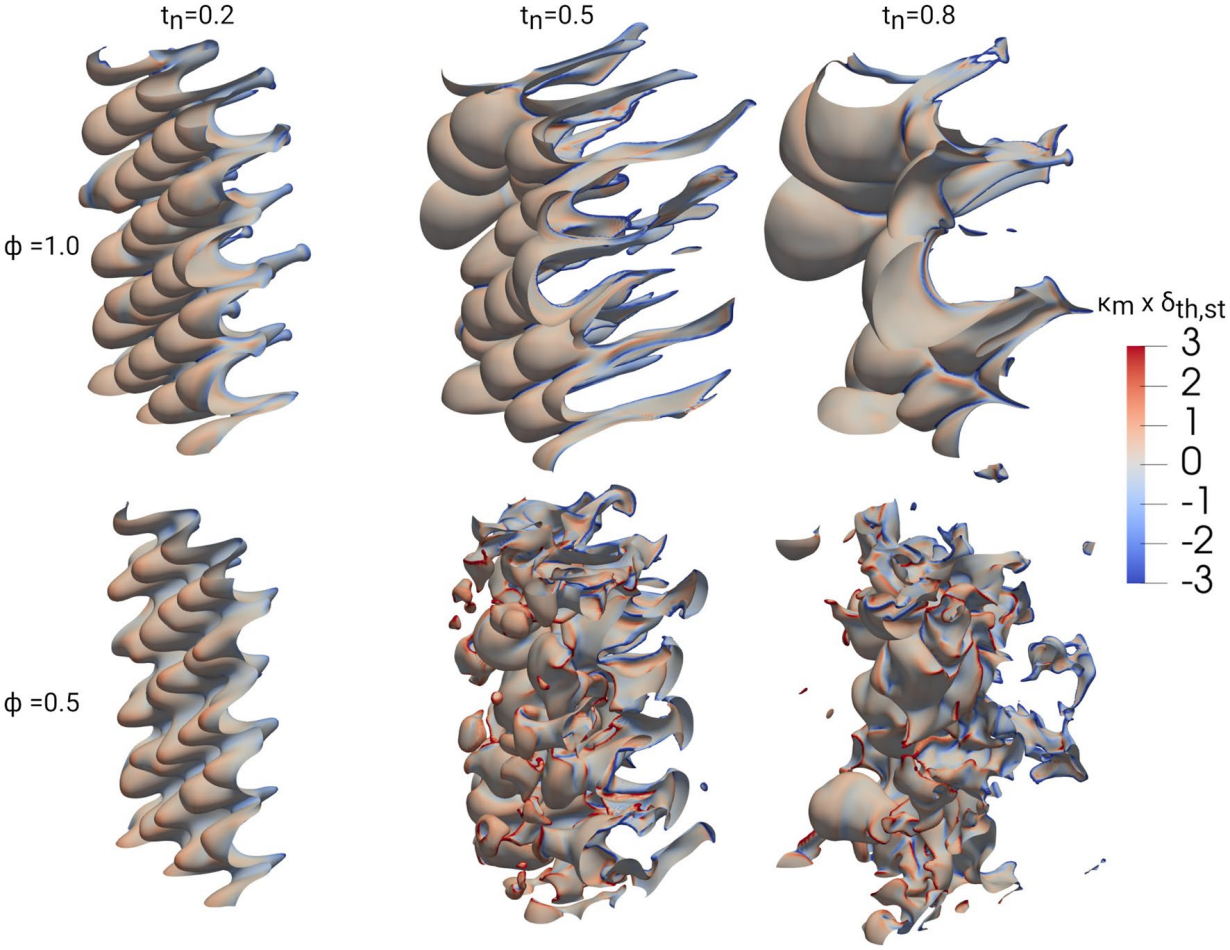
Isosurface of $c=0.5$, colored by $\kappa _{\text {m}} \times \delta _{\text {th,st}}$, for $\phi =1.0$ (top) and $\phi =0.5$ (bottom) for different normalized times $t_{\text {n}} = t \times S_{\text {L,st}}/\delta _{\text {th,st}}=0.2,0.5,0.8$ (after first shock, directly and later after reshock).

The FSI events can further be characterized by the local mean curvature $\kappa _{\text {m}}=0.5 \nabla \cdot (-\nabla c/|\nabla c|)=(\kappa _1 + \kappa _2)/2$ and the Gaussian curvature $\kappa _{\text {g}}=\kappa _1 \kappa _2$, where $\kappa _1$ and $\kappa _2$ denote the principal curvatures^29^. The distribution of FSI topologies on the $\kappa _m$-$\kappa _g$ plane is shown in Fig. 7. In order to interpret the figure it is important to understand the topologies which are characterized by the $\kappa$ values. The region of $\kappa _{\text {m}}<0$ ($\kappa _{\text {m}}>0$) corresponds to flame surfaces which are concave (convex) to the unburned reactants. Regions of $\kappa _{\text {g}}<0$ correspond to hyperbolic saddle-like topologies, whereas regions of $\kappa _{\text {g}}>0$ correspond to elliptical cup-like topologies. The boundary given by $\kappa _{\text {g}}>\kappa _{\text {m}}^2$ is marked by a black line in Fig. 7 and cannot be reached, as it would imply complex principal curvatures. The UPs are found in the region of $\kappa _{\text {g}}>0$ and $\kappa _{\text {m}}<0$ and can be associated with cup-like concave topologies, meaning regions of unburned gas surrounded by burned gas regions propagating inwards. The BPs are also found at $\kappa _{\text {g}}>0$ but in the region of $\kappa _{\text {m}}>0$ and are therefore associated with cup-like convex topologies, meaning regions of burned gas propagating outwards surrounded by regions of unburned gas. For cases with a high FSI count ($\phi =0.5$ and nonreactive after reshock), the tunnel-type topologies can be found in all regions of the $\kappa _{\text {m}}$-$\kappa _{\text {g}}$ plane, except the cup-like convex regime for TCs and cup-like concave regime for TFs. For $\phi =1.0$, all FSI events are associated with geometries concave to the unburned reactant ($\kappa _{\text {m}}<0$), meaning that they are situated on iso-surfaces propagating into the burned gas side of the flame (see Fig. 8). Again, the nonreactive case exhibits attributes of both the lean and stoichiometric cases. The main differences are the additional instances of FSI at $t\times S_{\text {L,st}}/\delta _{\text {th,st}}=0.2$ for $\kappa _{\text {m}}>0$, as the combination of decreased reactivity and increased wrinkling lead to the detection of additional self interactions on the convex flame cusps. A better understanding on how $\kappa _{\text {m}}$ is distributed across the flame surfaces can be gained from Fig. 8, showing isosurfaces at $c=0.5$ coloured by $\kappa _{\text {m}}$ throughout the different stages of the RMI and for $\phi =1.0$ and $\phi =0.5$ (nonreactive has been omitted for brevity). For $\phi =1.0$, the largest negative curvatures can be found on the narrow funnels extending into the burned gas, which is also the case where most FSI events were detected in Fig. 7. In contrast, the large convex flame bulges extending into the unburned gas lead to relatively low positive curvatures, which will make the detection of FSI in this regime unlikely. The effects of the small scale wrinkling and mixing after the reshock can be clearly seen for $\phi =0.5$ as both large positive and negative values of $\kappa _{\text {m}}$ can be found. These structures facilitate the build-up of burned and unburned tunnel and pocket type structures, as was shown in Fig. 7. The differences found in the topology distributions seen in Figs. 4 and 6 can also be attributed to the different flame structures developing in relation to the primary flow direction. For $\phi =1.0$, the flame structure is statistically asymmetric in regards to the burned (narrow funnels) and unburned (large cusps) sides of the flame front. This stands in contrast to the lean and nonreactive cases, showing a statistically symmetric flame structure, with small cusps/bulges and wrinkles appearing on both sides of the flame. The effects of the differences in flame thickness on the flame perturbation after the first shock-flame interaction can also be seen in Fig. 8, as the stoichiometric flame is distorted more than the lean flame at $t \times S_{\text {L,st}}/\delta _{\text {th,st}}=0.2$ leading to FSI in the flame locations which are concave towards the unburned reactants.

## Summary and conclusions

High-fidelity simulation data of shock-flame interactions of a planar shock wave interacting with a statistically planar ${\text {H}}_2$/air flame in a rectangular channel is used to study the temporal development and topology of flame self interactions (FSI) occurring due to RMI. The equivalence ratio $\phi$ is found to be a major influencing factor on the FSI as it affects the flame thickness and reactivity^17,18^. At early stages of the RMI, after the first shock flame interaction, a slight increase in FSI can be detected for the stoichiometric case ($\phi =1.0$), as its relatively thinner flame front allows for a higher amount of flame distortion compared to the lean ($\phi =0.5$) case. These interactions take place in narrow and concave (towards the unburned gas) flame funnels extending into the burned gas region. At later stages (after the reshock), the increased reactivity for $\phi =1.0$ prohibits the formation of small wrinkled structures, which keeps the number of FSI events small. For $\phi =0.5$, only a small number of FSI events is detected initially, due to the comparatively large flame thickness, which reduces the amount of distortion produced by the RMI. After the reshock, the normalized FSI frequency is about 10-20 times higher than for $\phi =1.0$ as the reduced reactivity enables larger amounts of small scale wrinkling and mixing of the flame. The eigenvalues of the Hessian Matrix of the reaction progress variable *c* have been used to determine the topological structure of the FSI. The predominant topologies in both cases are tunnel formation (TF) and tunnel closure (TC), with additional low amounts of unburned gas pocket (UP) and burned gas pocket (BP) being detected depending on the case. For $\phi =0.5$, burned and unburned gases are mixed in the wrinkled flame front rather symmetrically, leading to nearly equal amounts of burned and unburned pocket-type topologies and tunnel-type topologies, respectively. This is also shown by histograms of FSI topologies conditional on *c*, where the topologies are distributed rather uniformly with respect to *c*. In contrast, the statistically asymmetric flame structure at $\phi =1.0$ leads to an equally asymmetric distribution of topologies, with most FSI being detected at $c<0.5$. Characterization of the FSI structure using the local mean curvature and Gauss curvature, reveals further differences between the cases. For $\phi =1.0$, the interactions are primarily detected in the regions of the flame surface, which are concave to the reactants, whereas for $\phi =0.5$ at later stages, interactions can be found in both concave and convex regions. The results are further emphasized by comparing them with a nonreactive case, i.e. resembling the $\phi =1.0$ case with deactivated reactive source term. Initially, the results of the nonreactive case resemble the results obtained for the $\phi =1.0$ case, as the similarity in flame thickness and initial parameters dominate the development of the FSI. Later, after the reshock, the results resemble the lean case, as the effects of reduced reactivity become more important. In conclusion, the study shows that FSI can have a major influence on the characteristics of the flame-shock interaction. Especially regarding the development of surface-based closure models for lean mixtures, the effects of FSI should be taken into account for high-fidelity simulations of turbulent reactive RMI events.

## Methods

**Figure 9:**
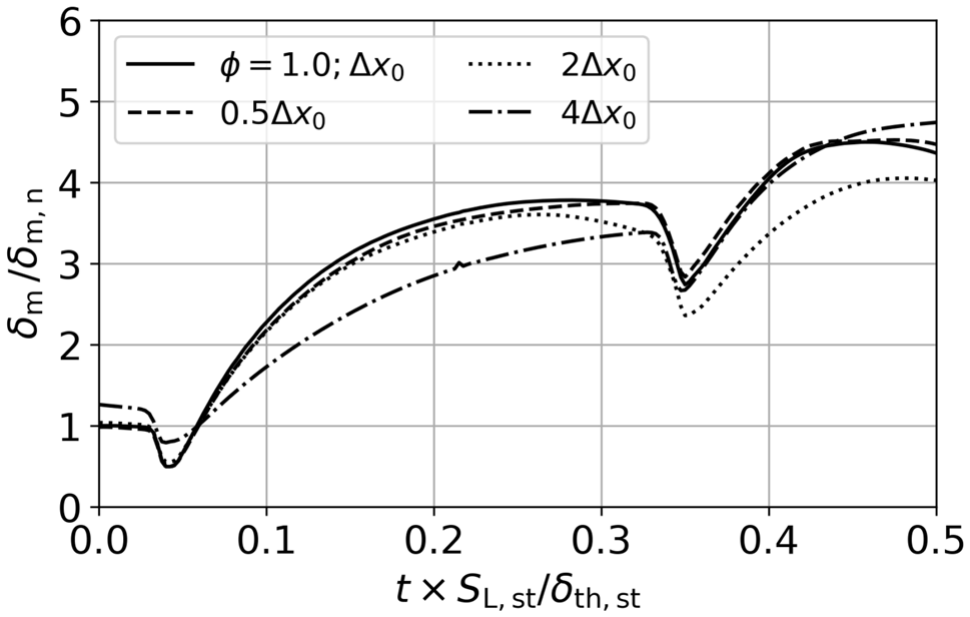
Convergence study of the normalized mixing width for $\phi =1.0$ at different resolutions.

A compressible 3D solver SENGA^30^ is used to conduct high-fidelity simulations of ${\text {H}}_2$/air shock-flame interactions. The compressible Navier–Stokes equations are solved in the non-dimensional form, including the balance equations of the total energy $e_{\text {t}}$ and the reaction progress variable *c*. With the product mass fraction $Y_{\text {p}}$, the reaction progress variable can be defined as $c=(Y_{\text {p}}-Y_{\text {p,0}})/(Y_{{\text {p}},\infty }-Y_{\text {p,0}})$, where subscripts 0 and $\infty$ refer to unburned conditions and chemical equilibrium, respectively. The equations of state are defined as

6 $$\begin{aligned} p&= \rho R_{\text {s}} T \end{aligned}$$

7 $$\begin{aligned} e_{\text {t}}&= c_{\text {v}}(T-T_{\text {ref}})+0.5 u_k u_k + H(1-c) ~, \end{aligned}$$

with the specific gas constant $R_{\text {s}}$, the specific isochoric heat capacity $c_{\text {v}}$ and the specific heat of reaction *H*. The thermophysical properties have been obtained using the Cantera-Toolkit^31^ for the respective equivalence ratios. To enable low-dissipation and oscillation free shock-capturing, a 5th order WENO-5 discretization scheme is utilized for spatial discretization^32^ in combination with a 3rd order Runge–Kutta scheme for time advancement^33^. As the flame wrinkling after shock-flame interactions is primarily controlled by fluid dynamic mechanisms (mostly baroclinic torque and vortex stretching^18^) rather than chemical kinetics, a one step Arrhenius-type approach is utilized to express the chemical source term ${\dot{\omega }}$. In this way the large computational costs encountered with detailed chemistry methods^34^ are avoided, while still capturing the effects of the reactivity on the RMI. Using the pre-exponential factor *B*, the Zeldovich number $\beta _{\text {z}}$ and the heat release parameters $\tau _{\text {h}}=(T_{\text {ad}}-T_0)/T_0$ and $\alpha _{\text {h}}=\tau _{\text {h}}/(1+\tau _{\text {h}})$, the source term ${\dot{\omega }}$ can be expressed as

8 $$\begin{aligned} {\dot{\omega }} = \rho B \exp \left[ -\frac{\beta _{\text {z}}}{\alpha _{\text {h}}}\right] (1-c) \exp \left[ \frac{-\beta _{\text {z}}(1-T^*)}{1-\alpha _{\text {h}}(1-T^*)} \right] \end{aligned}$$

where $\rho$, $T^*=(T-T_0)/(T_{\text {ad}}-T_0)$ and *c* denote the density, dimensionless temperature and reaction progress variable, respectively. The adiabatic flame temperature and the reference temperature corresponding to the initial state of the unburned $H_2/{\text{air}}$ gas-mixture are given by $T_{\text {ad}}$ and $T_0$. The pre-exponential factor *B* is adjusted using 1D steady state premixed flame simulations using Eq. (8), such that the desired laminar flame speed $S_{\text {L}}$ is achieved. When $B=0$, the *c*-transport equation is still solved, but the source term ${\dot{\omega }}=0$. By deactivating the source term, it is possible to separate the effects of reactivity (as measured by $S_{\text {L}}$) and flame thickness, providing important insights into the dominant parameters during the different phases of flame self-interaction (FSI). In the nonreactive case, the flame thickness and flame surface area should be interpreted as a interface thickness and interface area, respectively. The combustion properties ($\tau _{\text {h}}$, $T_{\text {ad}}$, $S_{\text {L}}$) of the hydrogen/air flame are calculated for the equivalence ratios $\phi =0.5$ (lean case) and $\phi =1.0$ (stoichiometric case) using the GRI-MECH 3.0 mechanism implemented in the Cantera toolkit^31^. While more specialized hydrogen-air mechanisms exist, the GRI-MECH 3.0 is a standard mechanism capable of modeling hydrogen-air oxidation with an accuracy more than sufficient for tuning a 1-step chemical model. The Atwood number is defined as $A_{\text {atw}}=(\rho _2-\rho _1)/(\rho _2+\rho _1)$, where $\rho _1$ and $\rho _2$ denote the densities of the light (burned) and heavy (unburned) gas, respectively. The pre-shock Atwood number is $A_{\text {atw}}=0.78$ for $\phi =1.0$ and $A_{\text {atw}}=0.69$ for $\phi =0.5$. An effective Lewis number ${\text {Le}}_{\text {eff}}$ of the mixture^35^ is calculated by blending the individual Lewis numbers (acquired from Cantera) for hydrogen ${\text {Le}}_{{\text {H}}_2}(\phi )$ and oxygen ${\text {Le}}_{{\text {O}}_2}(\phi )$ at the respective $\phi$ using the following equation valid for $\phi \le 1$

9 $$\begin{aligned} {\text {Le}}_{\text {eff}} = 1 + \frac{{({\text {Le}}_{{\text {O}}_2}(\phi ) -1)+({\text {Le}}_{{\text {H}}_2}(\phi ) -1)A_{\text {Le}}}}{1+A_{\text {Le}}} ~, \end{aligned}$$

where $A_{\text {Le}}=1+\beta _{\text {z}}({\text {max}}(1/\phi ,\phi )-1)$ and $\beta _{\text {z}}=5$ (see Bane et al.^36^ for detailed $\beta _{\text {z}}$ analysis). With the thermal laminar flame thickness of the stoichiometric case ($\phi =1$) being defined as $\delta _{\text {th,st}}=1/{\text {max}}|\nabla T^*|$, the domain of size $L_{\text {x}}\times L_{\text {y}} \times L_{\text {z}}= 128 \delta _{\text {th,st}} \times 32 \delta _{\text {th,st}} \times 16 \delta _{\text {th,st}}$ is uniformly discretized by $1024 \times 256 \times 128$ grid points, ensuring sufficient resolution of the inner flame structure and flame wrinkling ($\approx 8\Delta x_0$ within $\delta _{\text {th,st}}$^37^). A grid convergence study based on the normalized mixing width is shown in Fig. 9 (see Bambauer et al.^18^ for further details). The mixing width is calculated from $\delta _m = \int _0^{L_{\text {x}}}4\langle c \rangle (1- \langle c \rangle ) dx$, with $\langle c \rangle$ indicating averaging of the reaction progress variable *c* over the *y*-*z* plane. The normalization parameter is chosen as $\delta _{\text {m,n}}=\delta _{\text {m}}(t=0)$ for the reference ($\Delta x_0$) case. When reducing the base resolution ($\Delta x_0$) to $2\Delta x_0$ and $4 \Delta x_0$ small perturbations can no longer be resolved, leading to growing deviations of $\delta _{\text {m}}$. Further refinement to $0.5 \Delta x_0$ only has a marginal impact. For $A_{\text {atw}}=0.6$ to 0.7 and ${\text {Ma}}_{\text {s}} =1.5$ to 1.6, Weber et al.^38,39^ and Tritschler et al.^27^ estimate the smallest scales to be in the order of $\approx 10\Delta x_0$ for the late stages of the nonreactive RMI with multimode perturbation. Hence, the simulations can be considered as converged with respect to the qualitative analysis conducted in this work. A summary of the simulation parameters is given in Table 1.

**Table 1:** Simulation parameters for the stoichiometric (equivalence ratio $\phi =1.0$), lean ($\phi =0.5$) and nonreactive (nr) cases, with reference temperature $T_0$, reference pressure $p_0$, heat release parameter $\tau _{\text {h}}$, laminar flame speed $S_{\text {L}}$, effective Lewis number ${\text {Le}}_{\text {eff}}$, Zeldovich number $\beta _{\text {z}}$ and shock Mach number ${\text {Ma}}_{\text {s}}$.

	$\phi =1.0$	$\phi =0.5$	nr
$T_0$ (K)	298.15	298.15	298.15
$p_0$ (bar)	1	1	1
$\tau _{\text {h}}$	7.1	4.5	7.1
$S_{\text {L}}$ (m/s)	2.27	0.385	0
${\text {Le}}_{\text {eff}}$	1.1	0.5	1.1
$\beta _{\text {z}}$	5	5	-
${\text {Ma}}_{\text {s}}$	1.5	1.5	1.5

## Data Availability

The data that support the findings of this study are available from the corresponding author upon reasonable request.
